# A Cascaded Classification–Regression Framework for Shear Strength Prediction of Cold-Formed Steel Screw Connections

**DOI:** 10.3390/ma19122668

**Published:** 2026-06-21

**Authors:** Shen Liu, Rui Ren, Xiguang Liu, Zheng Luo

**Affiliations:** 1 Institute for Interdisciplinary Innovation Research, Xi’an University of Architecture and Technology, Xi’an 710055, China; liushen@xauat.edu.cn (S.L.); luo.zheng@xauat.edu.cn (Z.L.); 2 School of Civil Engineering, Xi’an University of Architecture and Technology, Xi’an 710055, China; renrui@xauat.edu.cn

**Keywords:** cold-formed steel, screw connection, machine learning, classification–regression, shear strength, failure mode

## Abstract

Existing AISI S100 provisions for cold-formed steel (CFS) screw connections lack codified strength equations for screw shear and net section fracture, and traditional machine learning (ML) models struggle to predict these minority failure modes due to imbalanced experimental datasets. This study proposes a cascaded ML framework that first classifies the failure mode and then predicts strength using mode-specific regressors. Two cascade strategies are evaluated: a Hard Classification Cascade (HC-C) and a novel Probability-Weighted Cascade (PW-C) that weights predictions by class probabilities to mitigate error propagation from misclassification. The predictive performance of the two cascaded models is benchmarked against a single regressor without classification. The superior PW-C model is then compared with AISI S100, and its resistance factor *ϕ* is subsequently calibrated in accordance with LRFD. Results show that the proposed cascaded models outperform the direct regression model, with PW-C improving the *R*^2^ for minority-class screw shear from 0.765 to 0.933 and for net section fracture from 0.784 to 0.912. Compared with AISI S100 provisions, PW-C extends coverage to the currently unaddressed failure modes and effectively captures screw group effects on shear strength based on a database of 564 tests. Reliability analysis yields an overall *ϕ*_c_ of 0.64 for the PW-C model, with a recommended divisor of 1.15 for direct application within the AISI design framework. This work provides a practical, data-driven pathway for updating design codes to cover failure modes beyond current specification limits.

## 1. Introduction

Cold-formed steel (CFS) structures enjoy widespread use in many countries and design specifications due to their light weight, high strength, ease of erection, and favorable seismic performance [1]. To extend the application of CFS shear wall structures to multi-story residential buildings, steel-sheathed and strap-braced CFS shear walls have been incorporated into the latest AISI S400 [2] and EN 1998-1-2 [3]. Experimental investigations and failure mode analyses [4,5,6], shaking table tests of multi-story structures [7,8], and discussions on design methods [9,10] have demonstrated that such shear walls possess excellent seismic capacity and are suitable for mid-rise buildings in seismically active regions.

In such systems, screw connections function as critical load-transferring components, and their shear strength and failure modes directly govern the structural safety. Experimental studies have shown that screw connections under shear loading can exhibit multiple failure modes, including bearing, screw shear, coupled tilting and pull-out, and net section fracture, each governed by fundamentally different load-carrying mechanisms and failure criteria [11]. Developing a unified design approach capable of accurately identifying the failure mode and predicting the corresponding shear strength is therefore essential for improving the design accuracy and reliability of CFS structures.

Since the 1990s, systematic experimental and theoretical investigations have established shear strength design formulas for CFS screw connections, with sheet thickness, steel grade, and screw count as the primary governing parameters, which have been instrumental to current design provisions [12,13,14]. More recently, research has increasingly focused on the coupled effects of screw arrangement, diameter, and end distance, identifying the group effect in multi-screw connections and the mechanisms governing failure mode transitions [15,16]. In parallel, the failure behavior of novel configurations such as corrugated sheet connections and screw–rivet hybrid systems has been characterized [17,18]. Additionally, the detrimental influence of elevated temperatures on failure modes, stiffness, and strength has motivated the development of strength reduction factors and fire design methods [19,20,21].

In recent years, machine learning (ML) and intelligent algorithms have offered a new paradigm for overcoming the modeling limitations of traditional empirical formulations. Feng et al. [22] used AdaBoost for RC column failure mode classification and capacity prediction, achieving high accuracy but without addressing classification uncertainty. Mangalathu et al. [23] employed RF with SHAP for failure mode prediction, focusing on interpretability rather than multi-failure mode regression. Existing studies on CFS structures have primarily focused on predicting the load-bearing capacity and failure modes of various CFS members, including built-up columns [24,25], stainless steel tubular columns [26], channel-section columns [27], and bolted connections [28,29]. All of these studies adopted a single-regressor approach, where a single model is trained to directly predict strength or classify failure mode. These studies consistently demonstrate that ensemble learning algorithms such as XGBoost (XGB) and Random Forest (RF) significantly improve prediction accuracy and systematically outperform current design specifications. However, when applied to datasets with imbalanced failure mode distributions, conventional single regression models exhibit a strong bias toward majority classes, leading to notably poor accuracy for minority failure modes. For instance, Feng et al. [22] reported that the AdaBoost-based classification precision for minority classes was only 75%, compared with 100% for the majority class in RC columns. Zhong et al. [30] further showed that direct regression models for concrete-filled steel tube columns achieved a coefficient of determination (*R*^2^) above 0.9 for the majority classes but dropped to as low as 0.274 for the minority class. To address this issue, researchers have introduced a classification–regression hard cascaded framework, in which the failure mode is first classified before invoking a mode-specific regressor for strength prediction, thereby effectively mitigating the accuracy degradation caused by class imbalance [30,31]. Nevertheless, such studies remain very limited for CFS screw connections, in which the failure modes are notably more diverse.

In summary, three main limitations exist in current code-based methods [32,33,34] and ML-based approaches for predicting the shear strength of screw connections:

- Incomplete coverage of failure modes and unsatisfactory predictive accuracy. The current specifications provide no strength calculation method for screw shear and net section fracture. For bearing and tilting failures within their scope, the mean test-to-predicted strength ratios range from 1.099 to 1.484 with coefficients of variation of 0.193–0.250, indicating substantial scatter and conservatism [35].
- Neglect of the group effect in multi-screw connections. Codes assume uniform load distribution among fasteners and exclude screw count, causing deviations up to 38% with a pronounced non-conservative trend as the number of screws increases [19].
- Error propagation due to misclassification in cascaded frameworks. Although cascaded frameworks mitigate class imbalance, their accuracy hinges on correct classification. A misclassification can invoke an incorrect regressor and introduce substantial bias, yet current hard cascades lack mechanisms to resolve this uncertainty.

To address the aforementioned research gaps, the principal contributions of this study are as follows. (1) A cascaded classification–regression prediction framework is proposed, incorporating two cascade strategies: a hard classification cascade (HC-C), which first identifies the failure mode before invoking the corresponding mode-specific regressor for strength prediction, and a probability-weighted cascade (PW-C), which computes a probability-weighted sum of the predictions from all mode-specific regressors using the class probability vector output by the classifier. The PW-C strategy is specifically designed to mitigate the error propagation arising from misclassification in the HC-C strategy when specimens lie near failure mode boundaries. (2) The predictive accuracy of the two cascaded models and a conventional single regressor is systematically compared across all failure modes, with particular attention to the improvement achieved for minority failure modes and the conditions under which each strategy performs best. (3) The proposed model is comprehensively benchmarked against the AISI S100 provisions, demonstrating its advantages in both failure mode coverage and the characterization of the screw group effect. A reliability analysis is further conducted following the Load and Resistance Factor Design (LRFD) to calibrate the resistance factor, and a strength reduction divisor is proposed for direct integration into the current design framework, thereby offering a standardized pathway for the practical application of data-driven methods in CFS structural design.

The remainder of this paper is organized as follows. Section 2 presents the proposed cascaded ML methodology. Section 3 describes the experimental database comprising 564 test results. Section 4 addresses failure mode classification. Section 5 examines shear strength prediction by comparing the two cascade strategies, a single regressor, and the AISI S100 provisions, followed by a reliability calibration. Section 6 summarizes the key findings.

## 2. Methodology

### 2.1. Proposed Cascaded Prediction Framework

**Figure 1:**
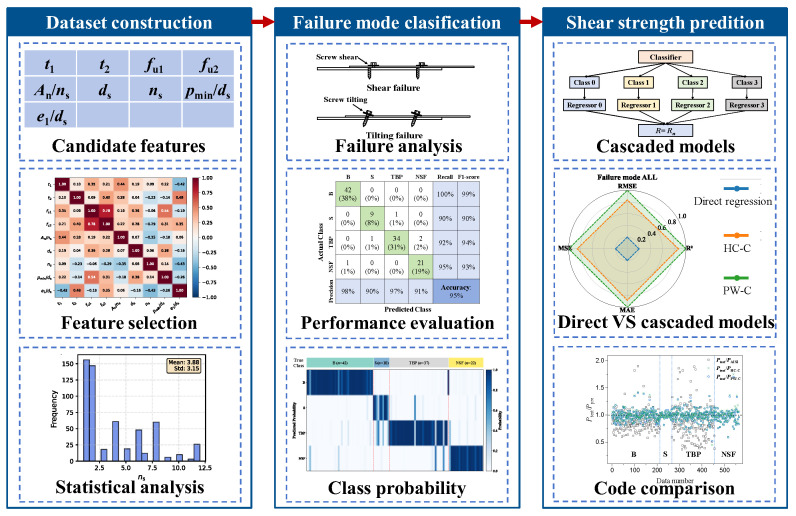
Proposed cascaded prediction framework.

This paper proposes a cascaded prediction framework that integrates a failure mode classification module with independent regression modules for each failure mode. Two strategies are introduced: the HC-C strategy and the PW-C strategy. Figure 1 illustrates the overall architecture of the framework, which comprises three stages: dataset preparation, failure mode identification, and shear strength prediction.

#### 2.1.1. Data Source

A specialized experimental database was established to support the training and testing of the ML models. The database comprises 564 single-shear test specimens of CFS screw connections, collected from 12 experimental studies published between 1998 and 2025. Detailed information on the database composition and parameter distributions is presented in Section 3.1.

#### 2.1.2. Failure Mode Classification

The failure mode prediction of the connections is performed using XGB and RF models, and the difference in prediction accuracy between the two is evaluated. In addition to the discrete class labels, both classifiers output the class membership probabilities for each specimen, represented as a probability vector *p* = (*p*_1_, *p*_2_, …, *p_k_*) that sums to one, where *k* denotes the number of failure modes. These probability outputs are obtained using the predict_proba method in scikit-learn [36]. For the RF model, the probability is computed as the mean of the predicted class probabilities across all decision trees in the ensemble. For the XGB model, the raw scores from all boosting rounds are aggregated and then converted into a probability distribution via the softmax function [37]. The predicted probabilities will serve as the weighting coefficients in the subsequent cascaded framework for shear strength prediction.

#### 2.1.3. Shear Strength Prediction

**Figure 2:**
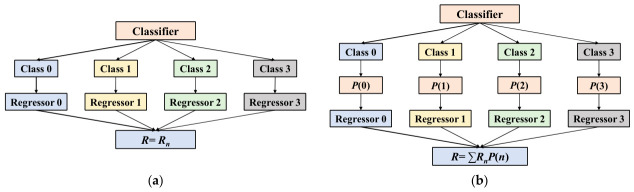
Cascade strategies: (**a**) HC-C and (**b**) PW-C.

According to different failure modes, the training set is divided into four subsets, and an independent regression model is trained for each failure mode. Subsequently, the failure mode classification model is cascaded with the failure mode-specific regression models in two ways (Figure 2):

- HC-C strategy

In this strategy, based on the discrete class label output by the classifier, only the regression model corresponding to the predicted failure mode is invoked for shear strength prediction. The final prediction is taken directly as the output of that model, i.e., *R* = *R_i_*, where *R_i_* is the predicted value from the regression model of the *i*-th failure mode.

- PW-C strategy

When a specimen lies in the transition region between failure modes, the classification confidence is relatively low, and relying solely on a single regression model may introduce considerable prediction bias. To overcome this limitation, the PW-C strategy is further proposed. This strategy utilizes the complete probability vector output by the classifier, invokes the regression models of all failure modes simultaneously, and computes the final shear strength as the probability-weighted sum of their individual predictions:

(1) $$R=\sum_{i=1}^{k}p_{i}R_{i}$$

To validate the effectiveness of the two cascaded frameworks, this study compares the predictions of the cascaded models with those of a direct regression method (a single regression model trained on the entire training dataset) and with calculations from existing design codes. The accuracy and reliability of the proposed methods in predicting the shear strength of screw connections are then evaluated.

### 2.2. ML Methods Selection

**Figure 3:**
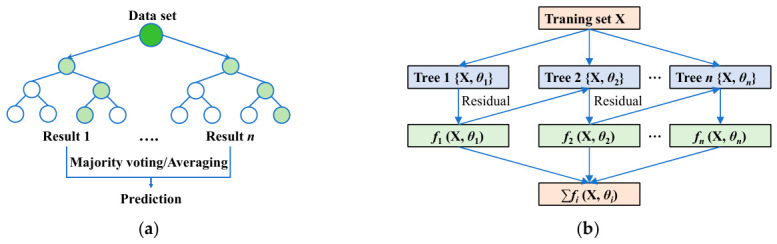
ML models diagram of (**a**) RF and (**b**) XGB.

RF [38] and XGB [37] represent the two mainstream paradigms in ensemble learning, namely bagging and boosting, which are complementary at the mechanistic level (Figure 3). The parallel training architecture of RF offers computational efficiency and robustness to hyperparameter deviations, whereas the sequential optimization strategy of XGB pursues higher prediction accuracy through iterative residual correction. Given that both models have demonstrated excellent generalization capability and possess complementary strengths, this study employs both for failure mode classification and systematically compares their predictive performance and probability output characteristics.

Compared with the entire dataset, the sample size of each failure mode subset is limited. The Bagging mechanism of RF, based on bootstrap sampling and ensemble averaging, provides an inherent regularization effect, which yields more robust predictive performance under small-sample conditions. In contrast, boosting methods are prone to overfitting the noise in the training data in such scenarios. Therefore, despite XGB’s potential for higher accuracy with abundant data, RF is more reliable for the limited and imbalanced failure mode-specific datasets. Consequently, the independent regression models for each failure mode within the cascaded framework are uniformly constructed using the RF algorithm.

### 2.3. Data Pre-Processing

To evaluate the generalization performance of the model, the 564 samples were randomly divided into a training set (452 samples) and a test set (112 samples) using an 80/20 split. Given the imbalanced distribution of failure mode categories in the database, a stratified sampling strategy was employed to ensure that the proportions of each category in the training and test sets remained consistent with those in the original dataset. To eliminate the influence of dimensionality, all input features were standardized using the Z-score normalization method, as expressed in Equation (2):

(2) $$x_{\mathrm{std}}=\frac{x-\mu }{\sigma }$$

where μ and σ are the mean and standard deviation (std) of each feature computed from the training set, respectively.

### 2.4. Hyperparameter Optimization

This study employed a 5-fold cross-validation strategy for hyperparameter tuning, wherein the training set was randomly partitioned into five non-overlapping subsets. In each fold, four subsets were used for training, and the remaining subset served as validation. This procedure was repeated five times, with the average validation score across all folds taken as the performance metric. A grid search method was adopted to systematically traverse the predefined hyperparameter space and identify the optimal configuration that maximised the cross-validation score. All tuning procedures were conducted exclusively on the training set, with the test set entirely withheld to ensure an unbiased assessment of model generalisation capability. Table 1 summarises the key hyperparameters and their search ranges for the ML algorithms employed in this study, and detailed parameter descriptions are available in the Scikit-learn documentation [36].

### 2.5. Performance Metrics

#### 2.5.1. Regression Metrics

Four statistical metrics, namely *R*^2^, root mean square error (RMSE), mean square error (MSE), and mean absolute error (MAE), were employed to evaluate the performance of the regression models. Their definitions are as follows:

(3) $$R^{2}=1-\frac{\sum_{i=1}^{n}{\left(y_{i}-\hat{y_{i}}\right)}^{2}}{\sum_{i=1}^{n}{\left(y_{i}-\bar{y}\right)}^{2}}$$

(4) $$\mathrm{RMSE}=\sqrt{\frac{1}{n}\sum_{i=1}^{n}{\left(y_{i}-\hat{y_{i}}\right)}^{2}}$$

(5) $$\mathrm{MSE}=\frac{1}{n}\sum_{i=1}^{n}{\left(y_{i}-\hat{y_{i}}\right)}^{2}$$

(6) $$\mathrm{MAE}=\frac{1}{n}\sum_{i=1}^{n}\left|y_{i}-\hat{y_{i}}\right|$$

where *n* is the total number of samples, *y_i_* is the actual strength of the *i*-th sample, $\hat{y_{i}}$ is the corresponding predicted value, and $\bar{y}$ is the average of the actual values.

These metrics evaluate model performance from complementary perspectives: *R*^2^ quantifies the overall goodness-of-fit of the model, RMSE and MSE reflect the penalty imposed on large errors, and MAE provides an intuitive measure of the average error. The combined use of these four metrics enables a comprehensive assessment of model accuracy and robustness in the shear strength prediction task.

#### 2.5.2. Classification Metrics

For the failure mode classification task, accuracy, F1-score, and confusion matrices were adopted as the primary evaluation metrics. Accuracy is defined as the ratio of correctly predicted samples to the total number of samples:

(7) $$\mathrm{Accuracy}=\frac{TP+TN}{TP+TN+FP+FN}$$

where *TP*, *TN*, *FP*, and *FN* denote true positives, true negatives, false positives, and false negatives, respectively.

The F1-score is defined as the harmonic mean of precision and recall, providing a balanced measure that accounts for both false positives and false negatives:

(8) $$F1=2\times \frac{\mathrm{Precision}\times \mathrm{Recall}}{\mathrm{Precision}+\mathrm{Recall}}$$

(9) $$\mathrm{Precision}=\frac{TP}{TP+FP}$$

(10) $$\mathrm{Recall}=\frac{TP}{TP+FN}$$

Precision and Recall are both important evaluation metrics for classification models, yet improving one often comes at the cost of the other. Relying solely on either metric cannot fully reflect model performance. By comprehensively considering both, the F1-score provides a more robust measure of classification performance across different failure modes.

The confusion matrix presents the correspondence between model predictions and actual classes. Rows represent true classes, while columns represent predicted classes. Diagonal entries indicate correctly classified samples, and off-diagonal entries reflect misclassifications. Analysis of the confusion matrix reveals which failure modes are easily confused, providing guidance for model optimization or feature engineering.

## 3. Dataset Construction and Feature Analysis

### 3.1. Dataset Overview

The database includes 564 experimental data points collected from 12 studies published between 1998 and 2025, with basic information summarized in Table 2. The contribution of each study ranges from 7 to 93 specimens, with no single source dominating the database. The sheet thickness spans 0.33–3.00 mm across sources with substantial overlap, forming a continuous parameter space that avoids isolated clusters and ensures satisfactory model generalization. The number of screws ranges from 1 to 14, covering both single-screw and multi-screw connections, which enables the investigation of group effects on shear strength. All specimens were subjected to the single shear test.

### 3.2. Design Parameters

**Figure 4:**
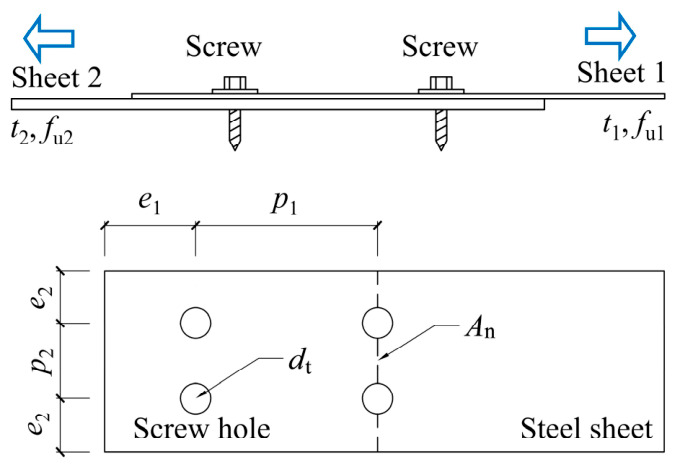
Parameters of typical screw connections.

A total of 11 parameters are considered for the ML models, encompassing both geometric configurations and material properties of screw connections. The geometric parameters include the thicknesses of the steel sheets in contact with (*t*_1_) and not in contact with (*t*_2_) the screw head, the screw diameter (*d*_s_), the number of screws (*n*_s_), the end distance (*e*_1_), the edge distance (*e*_2_), the screw spacings parallel (*p*_1_) and perpendicular (*p*_2_) to the loading direction, and the net cross-sectional area (*A*_n_). The material properties are represented by the tensile strengths of the two steel sheets (*f*_u1_, *f*_u2_). These parameters are selected based on their recognized influence on the shear strength and failure modes of screw connections in existing design codes and experimental studies. Schematic definitions are provided in Figure 4.

### 3.3. Failure Mode Analysis

**Figure 5:**
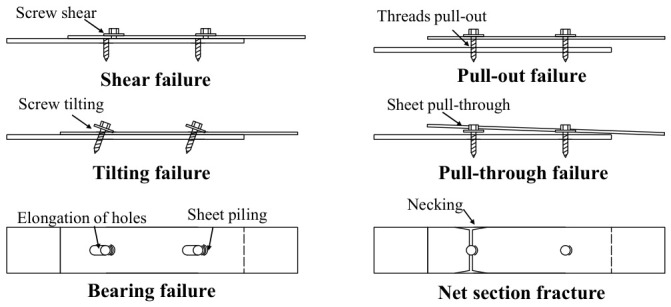
Typical failure modes of screw connections.

Typical failure modes of screw connections include screw shear, tilting, bearing, net section fracture, pull-out, and pull-through, as illustrated in Figure 5. However, in experimental tests, connections often exhibit mixed failure patterns, such as tilting combined with bearing, screw tilting accompanied by sheet bearing prior to shear fracture, and tilting simultaneously occurring with pull-out or pull-through. The observed failure modes were ultimately classified into four categories: bearing failure without significant screw tilting (B), screw shear (S), screw tilting accompanied by sheet bearing, pull-out or pull-through (TBP), and net section fracture (NSF). In the ML classification model, these failure modes are encoded as numerical labels [B: 0, S: 1, TBP: 2, NSF: 3] for model training. The corresponding sample sizes are 212 for B, 53 for S, 190 for TBP, and 109 for NSF.

### 3.4. Feature Selection

#### 3.4.1. Candidate Features

The shear strength per screw, *R*, and the failure mode are designated as the target features for the regression and classification models, respectively. Based on the design parameters, nine candidate input features are formulated. In addition to the basic design parameters *t*_1_, *t*_2_, *f*_u1_, *f*_u2_, *d*_s_, and *n*_s_, three dimensionless variables are introduced: *p*_min_/*d*, *e*_1_/*d*, and *A*_n_/*n*_s_. Here, *p*_min_ is the minimum of *p*_1_ and *p*_2_, which characterizes the screw spacing. The ratio *e*_1_/*d* quantifies the relative end distance, and *A*_n_/*n*_s_ serves as a key parameter for distinguishing net section failure from other failure modes.

In the classification task, all nine candidate features are adopted to identify the critical states of each failure mode. Feature subsets for the regression tasks are tailored to each failure mode, jointly informed by the governing physical mechanism and the available sample size to mitigate overfitting. For failure modes B and TBP, the failure mechanisms entail complex interactions among the screw, steel sheets, and geometric configurations. Therefore, the full set of nine candidate features is retained to fully capture the nonlinear interaction among parameters. For failure mode S, since its failure is concentrated in the fastener itself and the limited sample size, only four candidate features are selected, namely *t*_1_, *t*_2_, *d*_s_, and *n*_s_. For failure mode NSF, whose shear strength is dominated by the net section of the base material and geometric configurations, the candidate features are reduced to *f*_u1_, *A*_n_/*n*_s_, *n*_s_, and *p*_min_/*d*_s_.

#### 3.4.2. Feature Importance Analysis

**Figure 6:**
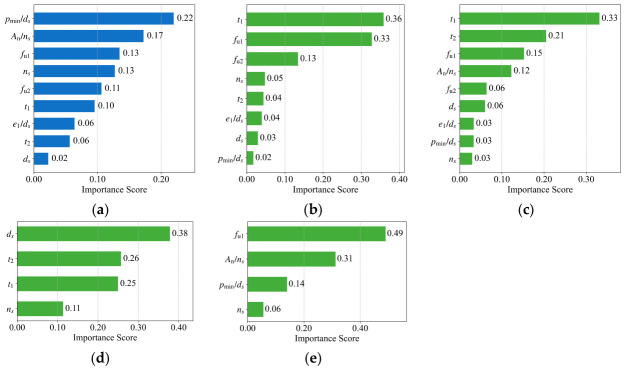
Feature importance for: (**a**) failure mode classification, and regression models for failure modes (**b**) B, (**c**) TBP, (**d**) S, and (**e**) NSF.

RF quantifies feature importance by averaging the impurity decrease contributed by each feature across all decision trees in the ensemble [38]. Figure 6 presents the feature importance for the classification model and the four regression models corresponding to each failure mode, respectively.

For the classification task, *p*_min_/*d*_s_ (0.22) and *A*_n_/*n*_s_ (0.17) achieved markedly higher importance scores than the remaining variables, indicating that screw spacing and net cross-sectional area are the key parameters governing failure mode identification. For the regression tasks, the dominant features varied distinctly across failure modes: *t*_1_ (0.36) and *f*_u1_ (0.33) for class B, *t*_1_ (0.33) and *t*_2_ (0.21) for class TBP, *d*_s_ (0.38) for class S, and *f*_u1_ (0.49) and *A*_n_/*n*_s_ (0.31) for class NSF. The above feature importance patterns are highly consistent with the design theory of CFS connections [33], which validates the rationality of the selected input features and the physical consistency of the proposed prediction framework. Furthermore, the candidate input features selected for different tasks all exhibit non-negligible importance, and no redundant features are observed.

#### 3.4.3. Feature Correlation Analysis

**Figure 7:**
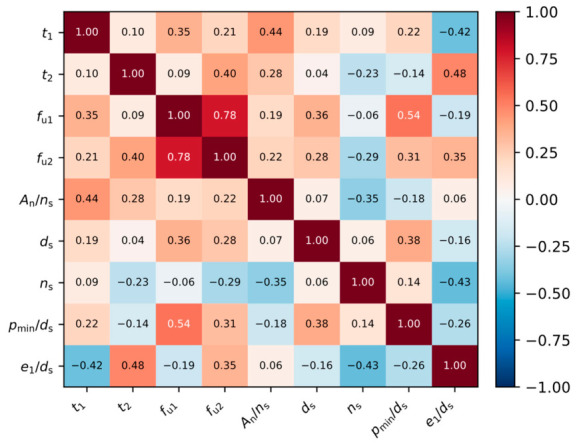
Correlation matrix heat map.

To investigate the linear correlations between the potential input features, the Pearson correlation coefficient matrix was calculated, as presented in Figure 7. The Pearson correlation coefficient *r* measures the strength of the linear relationship between two continuous variables, with values ranging from −1 to 1. The magnitude of *r* is interpreted as follows: $\left|r\right|\geq 0.7$ indicates a strong correlation, $0.4\leq \left|r\right|<0.7$ suggests a moderate correlation, $0.2\leq \left|r\right|<0.4$ represents a weak correlation, and $\left|r\right|<0.2$ implies a very weak or negligible correlation.

As can be observed from Figure 7, a strong correlation exists between *f*_u1_ and *f*_u2_ (*r* = 0.78), which is attributable to the fact that the two steel sheets in the connection tests were typically taken from the same batch or steel grade. Nevertheless, to accurately predict the connection performance under various combinations of sheet thickness and strength, both strength parameters are retained in this study.

Based on the above analysis, the input and target features employed in the regression and classification models are summarized in Table 3.

### 3.5. Statistical Distribution of Input Features

**Figure 8:**
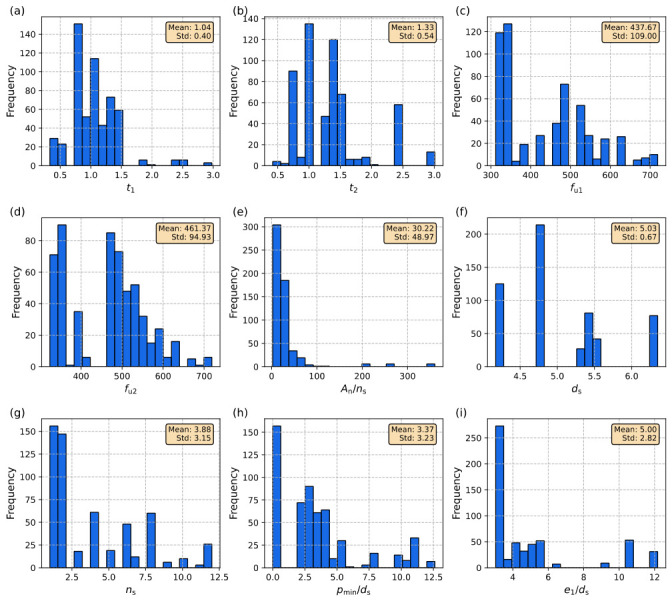
Statistical distribution of input features: (**a**) *t*_1_; (**b**) *t*_2_; (**c**) *f*_u1_; (**d**) *f*_u2_; (**e**) *A*_n_/*n*_s_; (**f**) *d*_s_; (**g**) *n*_s_; (**h**) *p*_min_/*d*_s_; (**i**) *e*_1_/*d*_s_.

The histograms of all input features for both the classification and regression models are presented in Figure 8. The vertical axis represents the frequency of occurrence, with the mean and std of each feature also annotated. As can be observed from Figure 8, *t*_1_ ranges from 0.33 mm to 2.98 mm, with a mean value of 1.04 mm, whereas *t*_2_ varies between 0.42 mm and 3.00 mm, corresponding to a mean of 1.33 mm. These statistics indicate that the database encompasses both thin and thick sheets, thereby providing a robust dataset for investigating connection performance under various sheet thickness combinations. Furthermore, the tensile strength of the sheet steel ranges from 312.1 MPa to 718.7 MPa, covering both ordinary and high-strength steels.

The mean value of *A*_n_/*n*_s_ is 30.22 mm^2^, with a notably high COV of 1.62, indicating significant dispersion. This variability is primarily attributed to the diverse combinations of cross-sectional dimensions and screw counts among the specimens. The diameter *d*_s_ of screws ranges from 4.17 mm to 6.35 mm, with a mean value of 5.03 mm, primarily covering screw sizes #8 to #14 commonly used in engineering practice. The number *n*_s_ of screws varies from 1 to 12, encompassing both single-screw and multi-screw connection specimens, thereby providing data support for investigating the group effect.

The dimensionless parameters *e*_1_/*d*_s_ and *p*_min_/*d*_s_ have mean values of 3.37 and 3.88, with a COV of 0.96 and 0.81, respectively. These statistics indicate a wide coverage of data, encompassing diverse screw spacing and edge distance configurations. It should be noted that the end distance, edge distance, and screw spacing of most specimens in the database satisfy the requirements for shear test specimens specified in the AISI S100 [33] and AS/NZS 4600 [34]. Overall, the above feature parameters exhibit broad distribution ranges and reasonable levels of dispersion, ensuring the adequacy of the dataset.

## 4. Failure Mode Identification

### 4.1. Performance Comparison

The classification performance of the RF and XGB models was evaluated using accuracy, F1-score, precision, recall, and confusion matrices. As summarized in Table 4, both models achieved excellent performance on the training set, with all metrics exceeding 0.96. On the test set, the two models yielded comparable overall performance. RF attained an accuracy, F1-score, precision, and recall of 0.946, while XGB achieved 0.955 for all four metrics. These results demonstrate that both models possess robust and similar generalization capabilities.

**Figure 9:**
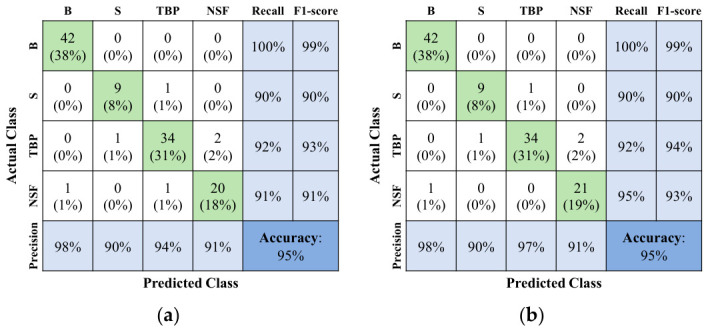
Confusion matrices for (**a**) RF, and (**b**) XGB model.

The confusion matrices of both models on the test set are presented in Figure 9. Class B is perfectly identified by both models, with a recall of 1.00 and a precision of 0.98, indicating highly distinct failure characteristics. For Class S, both models achieved identical recall and precision of 0.90, with the single misclassified sample erroneously predicted as TBP in both cases. For Class TBP, both models attained the same recall of 0.92, whereas XGB yielded a higher precision of 0.97 compared with 0.94 for RF, suggesting fewer false positives for this failure mode. For Class NSF, XGB outperformed RF in recall, 0.95 versus 0.91, at an equivalent precision of 0.91.

Overall, the two models exhibited comparable classification performance across all failure modes. Nevertheless, XGB demonstrated moderately superior per-class metrics on the critical failure modes, achieving higher precision for TBP and higher recall for NSF.

### 4.2. Probabilistic Classification Results

In addition to the discrete class labels, both classifiers output the predicted probabilities of each specimen belonging to the four failure modes, represented as a probability vector *p* = (*p*_B_, *p*_S_, *p*_TBP_, *p*_NSF_) that sums to one. These probability outputs serve as the weighting coefficients in the PW-C framework, where the final predicted shear strength is obtained as the probability-weighted sum of the individual predictions from all four failure mode-specific regression models.

**Figure 10:**
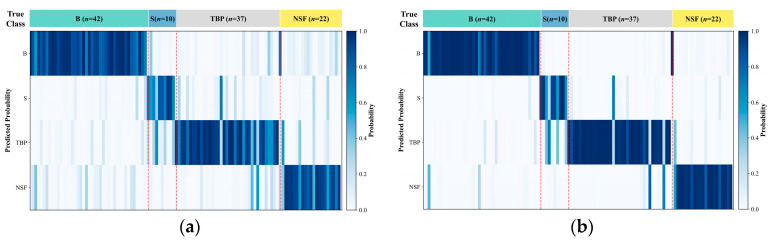
Probabilistic classification results on testing set of (**a**) RF and (**b**) XGB model.

Figure 10 presents the complete probability distributions of both models on the test set. Table 5 summarizes the distribution of the maximum predicted probability *p*_max_ for each specimen across different intervals.

As shown in Table 5 and Figure 10, XGB exhibits an overall higher prediction confidence than RF. Specifically, XGB assigns *p*_max_ ≥ 0.90 to 81.9% of the test specimens, markedly surpassing the 54.9% achieved by RF, which indicates that XGB can identify failure modes with greater certainty. The proportions of predictions with *p*_max_ in [0.70, 0.90) and [0.50, 0.70) for RF are 29.7% and 13.5%, respectively, compared with 11.7% and 6.3% for XGB. In addition, RF produces two predictions with *p*_max_ < 0.50, whereas no such case is observed for XGB.

Overall, XGB yields a higher mean probability assigned to the true class and produces no predictions with *p*_max_ < 0.50. For the PW-C framework, a highly concentrated probability distribution implies that the predicted shear strength is predominantly governed by the correct failure mode-specific regression model for the vast majority of specimens, thereby effectively reducing the prediction noise introduced by non-dominant failure mode regression models. Based on its marginally higher accuracy and more concentrated probability distribution, XGB was selected as the classifier for the cascaded framework. It should be noted that formal statistical testing was not performed; the observed advantage of XGB over RF is modest and should be interpreted as indicative rather than definitive.

## 5. Shear Strength Prediction

A direct regression model is first developed using the RF algorithm to serve as a baseline reference for the subsequent cascaded methods. Due to the imbalanced distribution of failure mode samples in the dataset, the direct regression model yields insufficient prediction accuracy on minority classes. To address this issue, independent RF regression models are trained for each of the four failure modes. Based on the XGB classifier established in Section 4, two cascaded strategies, HC-C and PW-C, are constructed. The direct regression and cascaded frameworks are then systematically compared in terms of prediction accuracy. Finally, the proposed cascaded methods are benchmarked against current design codes to evaluate both prediction accuracy and reliability.

### 5.1. Prediction Accuracy Comparison

**Figure 11:**
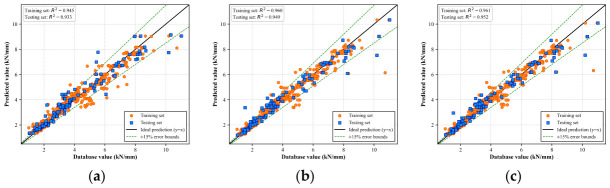
Comparison of shear strength predictions from (**a**) direct, (**b**) HC-C, and (**c**) PW-C models with experimental database results.

To systematically evaluate the predictive performance of each method, the direct regression model, the HC-C model, and the PW-C model were employed to predict *R*, and their predictive performance was quantitatively evaluated using *R*^2^, RMSE, MSE, and MAE. The performance metrics of the three methods on the test set are summarized in Table 6, and the scatter plots comparing predicted and experimental values are presented in Figure 11.

As shown in Table 6 and Figure 11, all three methods achieve an overall *R*^2^ above 0.93, and the results on the training and test sets are close, indicating that each method delivers satisfactory overall prediction performance with good generalization capability. However, the three methods differ substantially in prediction uniformity across failure modes. The direct regression model is heavily biased toward failure modes with abundant samples. The *R*^2^ values for Class B and Class TBP reach 0.956 and 0.916, respectively. In contrast, the minority classes S and NSF yield *R*^2^ values of only 0.765 and 0.784. Their RMSEs reach 0.794 kN and 0.825 kN, approximately three times that of Class B. This result suggests that a single regression model trained on the entire dataset tends to learn the dominant patterns of the majority classes and fails to adequately capture the bearing capacity relationships of the minority classes.

In contrast, both cascaded strategies significantly improve the prediction uniformity across failure modes. The HC-C model raises the *R*^2^ of Class S from 0.765 to 0.947, and that of Class NSF from 0.784 to 0.887. The PW-C model also improves the *R*^2^ of Class S to 0.933 and that of Class NSF to 0.912. Notably, for the majority classes B and TBP, the cascaded models maintain the prediction accuracy of the direct regression model without sacrificing performance on the majority classes. In terms of overall metrics, the PW-C model shows the best performance, achieving an *R*^2^ of 0.952, an RMSE of 0.518 kN, and an MAE of 0.342 kN across all specimens.

For Class B, the HC-C model performs best. This is because all Class B specimens are correctly identified by the classifier, and the HC-C model invariably invokes the correct failure mode-specific regression model, fully reflecting the advantage of the cascaded framework. For the other three failure modes, the classifier exhibits varying degrees of misclassification. Except for Class S, the PW-C model outperforms the HC-C model for both Class NSF and Class TBP. It should be noted that for Class TBP, the direct regression model achieves higher accuracy than the cascaded models, which can be attributed to two factors. First, the recall for Class TBP is 92%, with three misclassified specimens. The HC-C model invokes incorrect regression models for these specimens, and although the PW-C model mitigates this issue to some extent, it is still affected by incorrect weight allocation. Second, Class TBP has a relatively large sample size, and the direct regression model alone is already capable of learning its shear strength relationships reasonably well, leaving limited room for improvement through the cascaded strategies.

To further illustrate the behavior of PW-C and HC-C under misclassification, Table 7 presents five test specimens for which the classifier made an incorrect prediction. For the HC-C model, classification accuracy is the primary determinant of prediction quality. If the regressor corresponding to the misclassified failure mode yields a strength estimate close to the true experimental value, the prediction error remains limited, as observed in Specimens 1, 2, 3, and 5. However, when the regressor deviates substantially, HC-C exhibits significant bias. Specimen 4 exemplifies this case, in which the classifier misclassified TBP as NSF and the NSF-specific regressor underestimated the strength, yielding a *P*_test_/*P*_predict_ ratio of 0.45 and consequently a large HC-C error.

In contrast, the PW-C model can mitigate such error propagation, particularly when the failure mode with the second-highest predicted probability coincides with the true failure mode. For Specimen 4, PW-C assigned 26.83% weight to TBP (the true mode), improving the *P*_test_/*P*_predict_ ratio from 0.45 (HC-C) to 0.52. This indicates that PW-C may offer improved robustness for specimens near classification boundaries, provided that the correct class carries non-negligible probability mass and its corresponding regressor is reasonably accurate. Nevertheless, further validation with larger datasets is required to confirm this observation.

Overall, the cascaded framework effectively mitigates the performance degradation on minority classes caused by class imbalance in direct regression. Between the two cascaded strategies, the HC-C model performs best when the failure mode classification is accurate and the prediction confidence is high. When specimens lie near the classification boundary, the PW-C model may provide additional prediction robustness through its probability-weighted fusion mechanism.

### 5.2. Code Comparison

#### 5.2.1. Code Methods

To further evaluate the accuracy of the proposed cascade models in predicting the shear strength of screw connections, the predicted results are compared with those calculated by AISI S100. The specification adopts semi-theoretical and semi-empirical formulas based on bearing and tilting failure modes.

The nominal shear strength per screw (*P*_nv_) formulated in AISI S100 [33] is determined by Equations (11)–(13):

(11) $$P_{nv}=4.2\sqrt{t_{2}^{2}df_{u2}}$$

(12) $$P_{nv}=2.7t_{1}df_{u1}$$

(13) $$P_{nv}=2.7t_{2}df_{u2}$$

where *t*_1_ is the thickness of the member in contact with the screw head or washer, *t*_2_ is the thickness of the member not in contact with the screw head or washer, *f*_u1_ is the tensile strength of the member in contact with the screw head or washer, *f*_u2_ is the tensile strength of the member not in contact with screw head or washer, and *d* is the nominal fastener diameter.

For $t_{2}/t_{1}\leq 1.0$, *P*_nv_ shall be taken as the smallest of Equations (11)–(13), for $t_{2}/t_{1}\geq 2.5$, *P*_nv_ shall be taken as the smallest of Equations (12) and (13), for $1.0<t_{2}/t_{1}<2.5$, *P*_nv_ shall be calculated by linear interpolation between the above two cases. If the strength of the connection is governed by Equation (11), the limit state is tilting of the screw; if Equation (12) or (13) governs the strength, the limit state is bearing. When interpolation is used, the limit state is bearing and tilting [35].

#### 5.2.2. Comparison of PW-C Model with Code Methods

**Figure 12:**
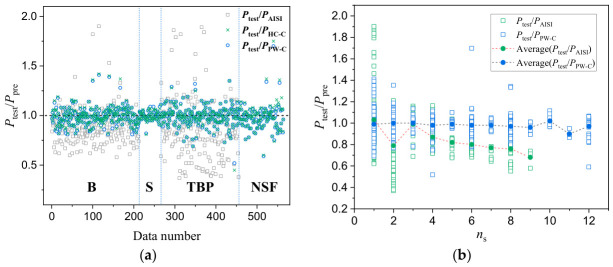
Comparison of *P*_test_/*P*_AISI_ and *P*_test_/*P*_PW-C_: (**a**) by failure mode, and (**b**) by *n*_s_.

Table 8 and Figure 12 compare the predictions of the cascade model PW-C with those obtained from the AISI S100 specification. Notably, the AISI S100 equations are applicable only to bearing and tilting failure modes. For screw shear failure, the nominal shear strength must be obtained from manufacturer-provided data, and the net-section capacity requires a separate calculation.

As shown in Table 8 and Figure 12a, for failure mode B, the PW-C model yields a mean *P*_test_/*P*_PW-C_ of 0.99 with a std of only 0.09, whereas the AISI specification gives a corresponding mean *P*_test_/*P*_AISI_ of 0.89 and a std of 0.17. For failure mode TBP, the PW-C model also achieves a mean *P*_test_/*P*_PW-C_ of 0.99 with a std of 0.11, while the AISI specification yields a mean *P*_test_/*P*_AISI_ of only 0.87 and a notably high std of 0.30. For failure modes S and NSF, which are not covered by the AISI specification, the PW-C model maintains high prediction accuracy, with mean *P*_test_/*P*_PW-C_ of 1.00 and 0.98 and stds of 0.05 and 0.12, respectively. The above results indicate that the AISI S100 design equations provide generally conservative predictions with considerable scatter, whereas the proposed PW-C model demonstrates significant advantages in prediction accuracy, scatter control, and failure mode coverage.

Figure 12b compares the AISI S100 and PW-C model predictions for specimens with varying numbers of screws. Data points for specimens that failed by net-section fracture or screw shear are excluded from the AISI S100 predictions, as these failure modes fall outside the scope of the specification. For single-screw connections, the average *P*_test_/*P*_AISI_ ratio is 1.03. As the number of screws increases, this ratio gradually decreases, reaching 0.68 for connections with nine screws. In contrast, the *P*_test_/*P*_PW-C_ ratio remains close to 1.00 irrespective of the number of screws, indicating that the *P*_test_/*P*_PW-C_ model effectively captures the adverse impact of group effect on shear strength.

In summary, the cascade model PW-C achieves significantly higher predictive accuracy and demonstrates the capability to predict shear strength across all potential failure modes, including failure NSF and S, while effectively accounting for the group effect of the screw.

#### 5.2.3. Reliability Analysis

A reliability analysis was conducted to further verify the accuracy and generalization ability of the proposed PW-C model and codified method within a load and resistance factor design (LRFD) framework. The resistance factor *ϕ* for the LRFD approach was calculated following the procedure outlined in Section K2.1.1 of AISI S100 [33]. A target reliability index *β*_0_ of 3.5 was adopted for connections. The calculation is expressed in Equation (14):

(14) $$\phi =C_{\phi }\left(M_{m}F_{m}P_{m}\right)e^{-\beta_{0}\sqrt{V_{M}^{2}+V_{F}^{2}+C_{P}V_{F}^{2}+V_{Q}^{2}}}$$

where:

$C_{\phi }$ is the calibration coefficient, taken as 1.52 for LRFD.

$M_{m}$ and *F*_m_ represent the mean values of material and fabrication factors, set to 1.1 and 1.0, respectively.

*V_M_* and *V*_F_ are coefficients of variation for material and fabrication factors, both equal to 0.1.

*V_Q_* denotes the coefficient of variation for load effects, taken as 0.21 for LRFD.

*C_P_* is the correction factor, calculated as *C*_P_ = (1 + 1/*n*) × *m*/(*m* − 2) where *n* is the number of tests and *m* is the degrees of freedom.

$P_{m}$ is the mean of the tested-to-predicted strength ratio (*P*_test_/*P*_pre_) for each method.

*V_P_* is obtained by dividing the standard deviation of the *P*_test_/*P*_pre_ ratios by *P*_m_.

As shown in Table 8, the resistance factor *ϕ*_c_ of the PW-C model for all failure modes is significantly superior to those provided by the AISI specification. For failure modes B and TBP, which are covered by AISI S100, the PW-C model achieves *ϕ* of 0.65 and 0.63, respectively, whereas the specification method yields only 0.49 and 0.32. Notably, *ϕ* of the specification for failure mode TBP is substantially lower than the LRFD resistance factor *ϕ*_AISI_ = 0.55 stipulated by AISI S100, rendering it inadequate for meeting reliability requirements. For failure modes S and NSF, which are not covered by the specification, the PW-C model achieves *ϕ* of 0.68 and 0.61, respectively. In the overall assessment across all specimens, the PW-C model yields a *ϕ* of 0.64, compared with only 0.40 for the AISI specification. These results demonstrate that the AISI S100 design equations, due to their considerable prediction scatter, yield considerably low resistance factors that limit design economy. In contrast, the PW-C model, with its higher prediction accuracy and lower dispersion, provides resistance factors that satisfy reliability requirements across all failure modes.

It is noteworthy that the overall resistance factor of the PW-C model (0.64) is higher than the LRFD resistance factor (0.55) stipulated by AISI S100. To align with the current code and facilitate direct adoption by designers, the predictions of the PW-C model require adjustment. To ensure that the adjusted resistance factor exactly equals 0.55, the required correction factor for the predicted strength is back-calculated using Equation (14), yielding a value of 1.15. Therefore, when the PW-C model is used for shear strength prediction in accordance with AISI S100, it is recommended that the model prediction be divided by 1.15 to obtain the nominal strength for design. This adjustment preserves consistency with current design practice while fully leveraging the superior prediction accuracy of the model.

## 6. Conclusions

This study proposed a cascaded ML framework for predicting the shear strength and failure modes of CFS screw connections, addressing the limitations of current AISI S100 provisions and conventional single regression approaches. Based on a database of 564 single-shear tests, the HC-C and PW-C strategies were systematically evaluated, benchmarked against AISI S100, and calibrated for reliability following LRFD. The main conclusions are as follows:

1. The cascaded framework offers a new solution to mitigate the class-imbalance problem. By decoupling failure mode classification from strength regression, it dramatically improves prediction uniformity across failure modes. For the minority classes S and NSF, the PW-C model improves *R*^2^ from 0.765 to 0.933 and from 0.784 to 0.912, respectively, compared with the direct regression model. This gain is achieved without sacrificing accuracy for the majority of classes.

2. Between the two cascaded strategies, the HC-C model performs best when failure mode classification is accurate and prediction confidence is high. When specimens lie near the classification boundary, the PW-C model tends to provide more balanced predictions across failure modes, potentially benefiting specimens near classification boundaries. Further investigation with larger datasets is needed to confirm this hypothesis.

3. The PW-C model extends predictive coverage to failure modes unaddressed by AISI S100 while capturing the screw group effect neglected by the code. For failure S and NSF, the model achieves mean *P*_test_/*P*_pre_ of 1.00 and 0.98 with stds of 0.05 and 0.12, respectively. Unlike the AISI S100 provisions, which become progressively unconservative as screw count increases, the PW-C model maintains a *P*_test_/*P*_pre_ close to 1.00 across all screw counts.

4. Reliability analysis following LRFD yields an overall *ϕ*_c_ of 0.64 for the PW-C model, substantially higher than the *ϕ* of 0.40 obtained for the AISI S100 provisions. A divisor of 1.15 is recommended for direct application of the model predictions within the AISI design framework, ensuring consistency with the code-specified *ϕ* = 0.55 while fully leveraging the model’s superior accuracy.

## Figures and Tables

**Table 1:** Hyperparameter tuning configuration.

Types	Models	Search Ranges and Optimal Values of Hyperparameters
Regressor	RF-B	n_estimators: [**100**, 150, 200]; max_depth: [5, 7, **10**]; min_samples_split: [5, **10**, 15]; min_samples_leaf: [**3**, 4, 5]; max_features: [sqrt, **log2**]
	RF-S	n_estimators: [30, **50**, 80]; max_depth: [3, **5**, 7]; min_samples_split: [**2**, 3, 4]; min_samples_leaf: [**2**, 3, 4]; max_features: [sqrt, **log2**]
	RF-TBP	n_estimators: [**50**, 100, 150]; max_depth: [7, 8, **9]**; min_samples_split: [**4**, 5, 6]; min_samples_leaf: [**2**, 3, 4]; max_features: [**sqrt**, log2]
	RF-NSF	n_estimators: [50, **80**, 100]; max_depth: [3, 4, **6**]; min_samples_split: [**4**, 5, 6]; min_samples_leaf: [**3**, 4, 5]; max_features: [**sqrt**, log2]
Classifier	RF	n_estimators: [**50**, 100, 200]; max_depth: [3, 5, **8**]; min_samples_split: [3, **6**, 9]; min_samples_leaf: [**2**, 3, 4]; max_features: [**sqrt**, log2]
	XGB	n_estimators: [50, 100, **200**]; max_depth: [3, **5**, **7**]; learning_rate: [0.01, **0.1**, 0.3]

The optimal values are bold.

**Table 2:** Summary of the screw connection shear test database.

Year	Source	No.	Screw Diameter (mm)	Screw Count	Sheet Thickness (mm)
1998	[39]	223	4.19, 4.72, 5.46	1–11	0.76–1.35
2006	[40]	79	4.80, 6.30	2–12	0.85–3.00
2012	[41]	11	4.17, 6.35	2–14	0.42–1.90
2013	[42]	24	5.35	1–14	1.20
2018	[43]	7	4.17, 4.82	1	0.90–1.50
2019	[44]	8	4.20	1	0.80–2.00
2019	[15]	18	5.35, 6.30	1	1.00–1.20
2020	[45]	33	4.80, 5.50, 6.30	1	0.75–2.98
2021	[46]	18	4.82, 5.49	1	1.48–2.48
2022	[47]	93	4.17, 4.82, 5.49	1	0.33–2.46
2022	[21]	12	4.76, 6.34	2	0.55–0.95
2025	[19]	38	4.80	1–12	1.50
	Total	564			

**Table 3:** Input and target features for classification and regression models.

		Input Features	Target Features
Classification models		*t*_1_, *t*_2_, *f*_u1_, *f*_u2_, *A*_n_/*n*_s_, *d*_s_, *n*_s_, *p*_min_/*d*_s_, *e*_1_/*d*_s_	(B, S, TBP, NSF)
Regression models	B	*t*_1_, *t*_2_, *f*_u1_, *f*_u2_, *d*_s_, *n*_s_, *p*_min_/*d*_s_, *e*_1_/*d*_s_	*P*
	S	*t*_1_, *t*_2_, *d*_s_, *n*_s_	
	TBP	*t*_1_, *t*_2_, *f*_u1_, *f*_u2_, *A*_n_/*n*_s_, *d*_s_, *n*_s_, *p*_min_/*d*_s_, *e*_1_/*d*_s_	
	NSF	*f*_u1_, *A*_n_/*n*_s_, *n*_s_, *p*_min_/*d*_s_	

**Table 4:** Classification performance of RF and XGB models.

Algorithm	Training Set				Test Set			
	Accuracy	F1-Score	Precision	Recall	Accuracy	F1-Score	Precision	Recall
RF	0.969	0.969	0.969	0.969	0.946	0.946	0.946	0.946
XGB	0.982	0.983	0.983	0.982	0.955	0.955	0.955	0.955

**Table 5:** Distribution of *p*_max_ intervals on the test set.

*p*_max_ Range	ML Model	
	XGB	RF
[0.90, 1.00]	91 (81.9%)	61(54.9%)
[0.70, 0.90)	13 (11.7%)	33 (29.7%)
[0.50, 0.70)	7 (6.3%)	15 (13.5%)
[0.00, 0.50)	0 (0.0%)	2 (1.8%)

**Table 6:** Performance metrics of direct and cascaded regression models.

Model	Direct Regression Model				HC-C Model				PW-C Model			
	*R* ^2^	RMSE (kN)	MSE	MAE (kN)	*R* ^2^	RMSE (kN)	MSE	MAE (kN)	*R* ^2^	RMSE (kN)	MSE	MAE (kN)
B	0.956	0.285	0.081	0.205	0.965	0.253	0.064	0.194	0.958	0.279	0.078	0.211
S	0.765	0.794	0.630	0.563	0.947	0.378	0.143	0.301	0.933	0.423	0.179	0.314
TBP	0.916	0.684	0.468	0.464	0.903	0.738	0.544	0.513	0.910	0.710	0.504	0.502
NSF	0.784	0.825	0.681	0.600	0.887	0.597	0.356	0.402	0.912	0.527	0.278	0.335
All	0.933	0.615	0.379	0.402	0.949	0.538	0.289	0.351	0.952	0.518	0.269	0.342

**Table 7:** Comparison of PW-C, HC-C, and direct regression for misclassified specimens.

No.	Failure Modes		*P*_test_/*P*_predict_				*p*_max_ (%)				*P*_test_/*P*_predict_		
	Test	Predicted	B	S	TBP	NSF	B	S	TBP	NSF	PW-C	HC-C	Direct
1	NSF	B	0.92	0.26	0.51	1.04	98.80	0.05	0.04	1.11	0.92	0.92	0.92
2	TBP	NSF	1.27	0.33	0.84	0.79	1.31	0.21	17.77	80.72	0.79	0.80	0.88
3	S	TBP	1.42	0.97	0.99	1.29	0.26	25.67	73.43	0.64	0.99	0.99	1.00
4	TBP	NSF	0.45	0.26	0.85	0.45	3.96	0.03	26.83	69.18	0.45	0.52	0.73
5	TBP	S	1.28	0.99	0.95	1.43	0.74	70.03	29.07	0.16	0.99	0.98	0.99

**Table 8:** Comparison between cascaded model predictions and AISI specification results.

Parameters	B (*n* = 212)		S (*n* = 53)		TBP (*n* = 190)		NSF (*n* = 109)		ALL (*n* = 402/564)	
	*P*_test_/*P*_AISI_	*P*_test_/*P*_PW-C_	*P*_test_/*P*_AISI_	*P*_test_/*P*_PW-C_	*P*_test_/*P*_AISI_	*P*_test_/*P*_PW-C_	*P*_test_/*P*_AISI_	*P*_test_/*P*_PW-C_	*P*_test_/*P*_AISI_	*P*_test_/*P*_PW-C_
Mean	0.89	0.99	-	1.00	0.87	0.99	-	0.98	0.88	0.99
Std	0.17	0.09	-	0.05	0.30	0.11	-	0.12	0.24	0.10
*ϕ*_c_	0.49	0.65	-	0.68	0.32	0.63	-	0.61	0.40	0.64

## Data Availability

The original contributions presented in the study are included in the article, further inquiries can be directed to the corresponding author.
